# Organic Extractives from *Mentha* spp. Honey and the Bee-Stomach: Methyl Syringate, Vomifoliol, Terpenediol I, Hotrienol and Other Compounds

**DOI:** 10.3390/molecules15042911

**Published:** 2010-04-22

**Authors:** Igor Jerković, Gordana Hegić, Zvonimir Marijanović, Dragan Bubalo

**Affiliations:** 1 Faculty of Chemistry and Technology, University of Split, N. Tesle 10/V, 21000 Split, Croatia; 2 Faculty of Agriculture, University of Zagreb, Svetošimunska 25, 10000 Zagreb, Croatia; 3 Marko Marulić Polytechnic in Knin, P. Krešimira IV 30, 22300 Knin, Croatia

**Keywords:** *Mentha* spp. honey, bee-stomach, headspace solid-phase microextraction (HS-SPME), ultrasonic solvent extraction (USE), gas chromatography and mass spectrometry (GC and GC/MS)

## Abstract

The GC and GC/MS analyses of the solvent organic extractive from the stomach of the bees, having collected *Mentha* spp. nectar, revealed the presence of methyl syringate (6.6%), terpendiol I (5.0%) and vomifoliol (3.0%) that can be attributed to the plant origin. Other major compounds from the bee-stomach were related to the composition of cuticular waxes and less to pheromones. Organic extractives from *Mentha* spp. honey were obtained by solvent-free headspace solid-phase microextraction (HS-SPME) and ultrasonic solvent extraction (USE) and analyzed by GC and GC/MS. The major honey headspace compounds were hotrienol (31.1%–38.5%), 2-methoxy-4-methylphenol (0.5–6.0%), *cis*- and *trans*-linalool oxides (0.9–2.8%), linalool (1.0–3.1%) and neroloxide (0.9–1.9%). Methyl syringate was the most abundant compound (38.3-56.2%) in the honey solvent extractives followed by vomifoliol (7.0–26.6%). Comparison of the honey organic extractives with the corresponding bee-stomach extractive indicated that methyl syringate and vomofoliol were transferred to the honey while terpendiol I was partially transformed to hotrienol in ripened honey.

## 1. Introduction

The chemical composition of the organic extractives is of great interest for characterizing the floral and/or geographical origin of honeys. In recent years a range of extractable natural organic substances that appear characteristic to be of the plant source have been identified as phytochemicals in different honeys [1,2]. For these analysis appropriate extraction methods should be applied to prevent the formation of artifacts generated by Strecker degradation, Maillard or non-enzymatic browning reactions [3]. In most cases, the extraction of organic compounds from the honey matrix is performed using solvents at room temperature [4,5], but the use of solvent-free systems (e.g. solid-phase microextraction) has also been proposed [6]. 

A broad range of aliphatic, aromatic, and/or degraded carotenoid-like structures have been reported in the honey extracts and headspace [1,2]. Unifloral honeys possess distinctive flavors, mainly derived from their nectar sources, indicating the presence of characteristic volatile components. The main components in source specific honey volatiles belong, in general, to three principal categories: terpenes, norisoprenoids, and benzene derivatives [1,2]. Some of these substances have been described as characteristics of the floral source, and other compounds, like some alcohols, branched aldehydes, and furan derivatives, may be related to the microbial purity of honey processing and storage conditions. Specific markers of the botanical origin were also found such as methyl anthranilate for citrus honey [7], *trans*-oak lactone for holm-oak honeydew [8] or 2-amino- and 4-aminoacetophenone for chestnut honey [9]. 

*Mentha*, the genus of Lamiaceae family, includes 25 species that are spread all over the world. Among them, *Mentha pulegium* L. and *Mentha aquatica* L., are well known aromatic plants. The constituents of *M. pulegium* L. oil have been studied [10,11] and a difference in the composition of the oil major components (pulegone, isomenthone and piperitone) depending on the region of cultivation in different countries have been found. The essential oil of *M. aquatica* L. is characterized by domination of menthofuran [12]. In general, very few references can be found on *Mentha* spp. honeys [13,14], but without any chemical composition data. As Lamiaceae pollen is under-represented in the spectra, the minimum pollen percentage for *Mentha* spp. honeys has been established at 20% [15,16].

There are only a few studies in which the organic extractives of the bee-stomach have been correlated with those of the corresponding honey. Most recently, the comparison of the components of the extracts of linden honey and bee-stomach showed that nectar and honey stomach contain many aldehydes which were found as corresponding acids in the honey [17]. To the best of our knowledge, organic extractives from *Mentha* spp. honey and from the corresponding bee-stomach have not yet been studied. A two-way approach was used for the research of honey volatiles: solvent-free headspace solid-phase microextraction (HS-SPME) and ultrasonic solvent extraction (USE). Isolated organic extractives were analyzed by gas chromatography and mass spectrometry (GC and GC/MS).

## 2. Results and Discussion

The gathered nectar is stocked in the bee-stomach, which can contain up to 60 μL of liquid, and the honey sacs containing *Mentha* spp. pollen grains were investigated. The enzymes in the salvia degrade nectar sucrose into glucose and fructose and cleave the glycosides. On returning to the hive, the content of the stomach is regurgitated into the waxy honeycomb and is ripened into the honey. Under the highly oxidative atmosphere of the honeycomb sensitive honey organic compounds can undergo oxidation [17]. The content of the stomach of the bees caught at the entrance of the hive on their way back from *Mentha* spp. nectar gathering was isolated by USE and compared to the extractives obtained from the ripe honey with focus on determination of plant derived compounds. The unifloral botanical origin of all the honey samples was confirmed by pollen analysis (up to 49% of *Mentha* spp. pollen dominated by *Mentha aquatica* L. and *Mentha pulegium* L.) according to recommended minimal values [15,16]. Identified accompanying pollen grains were from *Amorpha fruticosa*, *Centaurea* spp., Asteraceae and Brassicaceae with minor percentages of pollen from other species. 

The results presented below indicate great variability of the honey organic extractives composition strongly depending on the isolation method. Solvent-free HS-SPME method selectively isolated the honey headspace organic extractives dominated by high volatile compounds that were present with minor percentages in the honey solvent extracts. The medium-low volatile and relatively high polar organic compounds were the most abundant in the solvent extracts with limitations observed in the headspace composition. Both organic extractives were comprehensive and reliable for characterization of this honey without the presence of thermal derived artifacts.

### 2.1. Organic Extractives Isolated by Ultrasonic Solvent Extraction from the Bee-Stomach and Honey

**Figure 1:**
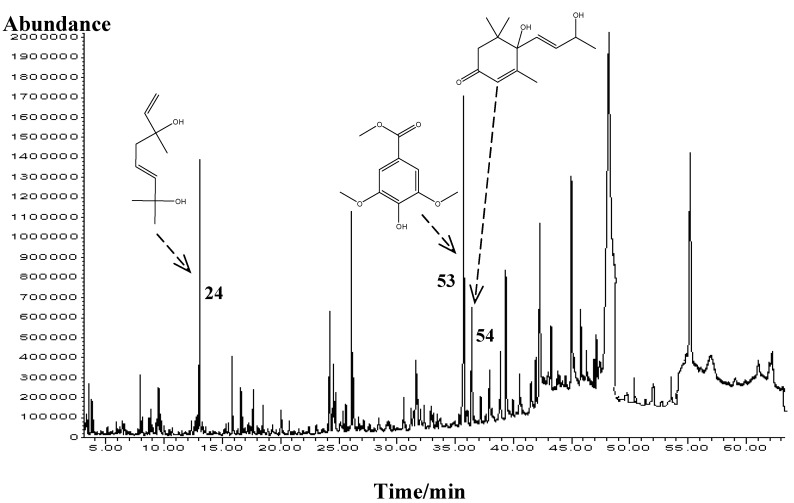
Representative TIC chromatogram of the bee-stomach organic extractive.

The content of the stomach of 70 bees caught at the entrance of the hive on their way back from *Mentha* spp. nectar gathering was isolated by USE and analyzed (a representative chromatogram is presented in Figure 1). The bee-stomach organic extract was mainly composed of the following groups of natural compounds (Table 1): fatty acids [(*Z*)-octadec-9-enoic acid (30.4%), hexadecanoic acid (6.4%)], fatty alcohols [(*Z*)-octadec-9-en-1-ol (5.7%), hexadecan-1-ol (3.3%), octadecan-1-ol (1.9%)] as well as higher aliphatic hydrocarbons [tetracosane (9.0%), heneicosane (0.7%)]. These chemical structures are particularly related to the composition of cuticular waxes and less to pheromones. The cuticular waxes contain aliphatic compounds from C_18_ to C_54_ dominated by hydrocarbons [18]. "Queen's pheromone", a well-equilibrated cocktail of fatty acids and aromatic compounds, is one of the most important sets of pheromones in the bee hive [19]. Only several structurally-related compounds of queen retinue pheromone were found in bee-stomach such as hexadecan-1-ol and different fatty acids. The major identified terpene in the bee-stomach was 3,7-dimethylocta-1,5-dien-3,7-diol, terpendiol I (5.0%) originated probably by glycosidase activity (cleavage of the nectar glycosides) in the salvia. This activity was already confirmed by the appearance of new monoterpenic alcohols (three linalool derivatives: 3,7-dimethylocta-1,5-dien-3,7-diol, 3,7-dimethylocta-1,6-dien-3,5-diol and 2,6-dimethyl-6-hydroxyocta-2,7-dienal) in the extract of the liquid isolated from the bee stomach having collected linden nectar [17]. Monoterpenic alcohol 3,7-dimethylocta-1,5-dien-3,7-diol, terpendiol I was found in *Mentha* spp. honey solvent extracts, but also can transform to hotrienol, the most abundant compound in the honey headspace, Table 2. Among all identified compounds, methyl syringate (6.6%) and vomifoliol (3.0%) can be considered plant derived phytochemicals in the bee-stomach having collected nectar from *Mentha* spp. flowers.

**Table 1:** Organic extractives composition from *Mentha* spp. honey and the bee-stomach.

No.	Compound	RI	Area percentage (%)										
			A					B					C
			Min.	Max.	Av.	SD.		Min.	Max.	Av.	SD.		
1.	2-Methylbutanoic acid	< 900	0.0	0.1	0.07	0.06		-	-	-	-		-
2.	2-Furanmethanol	< 900	0.1	0.2	0.13	0.06		0.0	0.2	0.10	0.10		-
3.	4-Methyloctane	< 900	-	-	-	-		-	-	-	-		0.2
4.	1,4-Dimethylbenzene	< 900	0.0	0.1	0.07	0.06		-	-	-	-		0.6
5.	Heptan-2-one	< 900	-	-	-	-		-	-	-	-		0.3
6.	1-(2-Furanyl)-ethanone	914	0.0	0.1	0.03	0.06		-	-	-	-		-
7.	Benzaldehyde^a^	965	0.0	0.1	0.03	0.06		-	-	-	-		-
8.	(*Z*)-Hex-3-enoic acid	1013	0.1	0.3	0.20	0.10		-	-	-	-		-
9.	Limonene^a^	1036	0.0	0.1	0.03	0.06		-	-	-	-		-
10.	Benzyl alcohol^a^	1037	0.1	0.5	0.30	0.20		0.0	0.5	0.27	0.25		-
11.	Phenylacetaldehyde^a^	1048	0.0	0.1	0.07	0.06		-	-	-	-		-
12.	5-Methylundecane^*^	1067	-	-	-	-		-	-	-	-		0.8
13.	*trans*-Linalool oxide (furan type)	1076	0.0	0.1	0.07	0.06		-	-	-	-		-
14.	4,5-Dimethyl-2-formylfuran	1078	0.0	0.2	0.10	0.10		0.0	0.2	0.10	0.10		-
15.	Methyl 2-furoate	1084	0.1	0.7	0.47	0.32		0.2	0.9	0.63	0.38		-
16.	Undecane^a^	1100	-	-	-	-		-	-	-	-		0.2
17.	Hotrienol	1106	0.2	0.6	0.47	0.23		0.1	0.6	0.43	0.29		-
18.	2-Phenylethanol^a^	1116	0.1	0.4	0.30	0.17		0.2	0.4	0.33	0.11		-
19.	5-Hydroxy-2-methyl-4H-Pyran-4-one	1139	0.0	0.2	0.10	0.10		0.0	0.1	0.07	0.06		-
20.	2,3-Dihydro-3,5-dihydroxy-6-methyl-4H-pyran-4-one	1145	0.0	0.2	0.10	0.10		0.0	0.2	0.13	0.11		-
21.	Octanoic acid^a^	1174	-	-	-	-		-	-	-	-		0.4
22.	Benzoic acid^a^	1162	0.4	1.3	0.97	0.49		0.2	0.9	0.63	0.38		-
23.	3,5-Dihydroxy-2-methyl-4H-pyran-4-one	1189	0.0	0.2	0.10	0.10		-	-	-	-		-
24.	3,7-Dimethylocta-1,5-dien-3,7-diol (terpendiol I)	1191	1.0	4.9	3.17	1.98		0.7	2.5	1.57	0.90		5.0
25.	Dodecane^a^	1200	-	-	-	-		-	-	-	-		0.8
26.	5-Hydroxymethyl-furfural	1230	0.1	2.7	1.60	1.35		0.2	5.1	2.97	2.51		-
27.	(*E*)-Dec-2-enal	1268	-	-	-	-		-	-	-	-		1.2
28.	Phenylacetic acid^a^	1269	1.0	7.7	3.83	3.44		0.3	5.1	2.37	2.47		-
29.	Nonanoic acid^a^	1273	-	-	-	-		-	-	-	-		0.3
30.	4-Acetylanilline	1308	0.0	0.2	0.10	0.10		-	-	-	-		-
31.	3-Hydroxy-4-phenyl-butan-2-one	1354	0.8	1.9	1.33	0.55		0.6	1.2	0.83	0.32		-
32.	Phenylpropanoic acid^a^	1361	0.0	0.6	0.27	0.31		0.0	0.3	0.17	0.15		-
33.	1-Hydroxylinalool	1365	0.1	0.4	0.23	0.15		0.2	0.3	0.20	0.10		-
34.	2-Phenylacetamide	1393	0.0	0.1	0.07	0.06		0.0	0.5	0.23	0.25		-
35.	2-Aminobenzoic acid	1416	0.0	0.8	0.33	0.42		0.0	0.7	0.27	0.38		-
36.	4-Hydroxybenzyl alcohol	1426	0.1	0.2	0.17	0.06		-	-	-	-		-
37.	2,6-bis(1,1-Dimethylethyl)-cyclohexa-2,5-diene-1,4-dione	1473	-		-	-		-	-	-	-		2.3
38.	2,6-di(1,1-Dimethylethyl)-4-hydroxy-4-methyl-cyclohexa-2,5-dien-1-one	1841	-	-	-	-		-	-	-	-		1.3
39.	2,6-di(1,1-Dimethylethyl)-4-metylene-cyclohexa-2,5-diene-1-one	1845	-	-	-	-		-	-	-	-		1.0
40.	Acetylvanillone	1494	0.0	0.3	0.17	0.15		0.0	0.3	0.17	0.15		-
41.	Pentadecane^a^	1500	-	-	-	-		-	-	-	-		0.3
42.	2,4-bis(1,1-Dimethylethyl)phenol	1525	-	-	-	-		-	-	-	-		1.2
43.	Methyl vanillate^a^	1527	0.4	1.3	0.83	0.45		0.3	0.8	0.60	0.26		-
44.	4-Hydroxybenzoic acid^a^	1558	0.3	0.9	0.67	0.32		0.0	0.2	0.10	0.10		-
45.	Vanillic acid^a^	1566	0.2	0.8	0.50	0.30		0.1	0.3	0.20	0.10		-
46.	3-Hydroxy-β-damascone	1617	0.0	0.5	0.27	0.25		0.1	0.3	0.20	0.10		-
47.	Isopropyl-pseudocumene^**^	1657	0.3	1.8	1.13	0.76		0.5	1.7	1.07	0.60		0.6
48.	Menthofuran	1661	0.0	0.4	0.20	0.20		0.0	0.4	0.17	0.21		-
49.	Syringaldehyde	1662	0.0	0.1	0.07	0.06		0.0	0.2	0.10	0.10		-
50.	Isopropyl-pseudocumene^**^	1678	0.0	1.8	1.03	0.930		0.5	1.7	1.07	0.60		-
51.	8-Hydroxyquinoline	1720	-	-	-	-		0.4	1.0	0.63	0.32		-
52.	4-Ethybenzen-1,3-diol	1743	0.3	0.4	0.37	0.06		0.2	0.7	0.40	0.26		-
53.	Methyl syringate^a^	1744	47.9	56.2	52.30	4.17		38.3	48.6	43.57	5.15		6.6
54.	Vomifoliol	1802	7.0	10.3	8.27	1.78		16.9	26.6	20.43	5.36		3.0
55.	3-Ethylbenzophenone	1826	-	-	-	-		-	-	-	-		0.6
56.	3-(4-Hydroxy-phenyl)-prop-2-enoic acid	1831	0.4	0.8	0.67	0.23		-	-	-	-		-
57.	Neophytadiene	1849	-	-	-	-		-	-	-	-		1.5
58.	Ferulic acid^a^	1867	0.0	0.2	0.10	0.10		-	-	-	-		-
59.	Diisobuthyl phtalate	1869	0.1	0.2	0.13	0.06		0.2	0.3	0.23	0.06		1.5
60.	Hexadecan-1-ol^a^	1882	0.5	1.0	0.70	0.26		0.9	1.2	1.03	0.15		3.3
61.	Nonadecane^a^	1900	-	-	-	-		-	-	-	-		0.3
62.	Hexadecanoic acid^a^	1963	0.8	6.7	2.97	3.25		0.7	1.8	1.30	0.56		6.4
63.	(*Z*)-Octadec-9-en-1-ol^a^	2060	1.0	1.6	1.27	0.31		1.3	2.4	1.93	0.57		5.7
64.	Octadecan-1-ol^a^	2084	0.2	0.3	0.27	0.06		0.1	0.6	0.37	0.25		1.9
65.	Heneicosane^a^	2100	-	-	-	-		-	-	-	-		0.7
66.	(Z)-Octadec-9-enoic acid^a^	2147	0.3	1.5	0.80	0.62		0.6	2.0	1.13	0.76		30.4
67.	(*Z*)-Tricos-9-ene	2330	-	-	-	-		-	-	-	-		2.2
68.	Tetracosane^a^	2400	0.7	2.4	1.30	0.95		0.8	2.0	1.50	0.62		9.0

A = *Mentha* spp. honey solvent extract with pentane and diethyl ether (1:2, v/v); B = *Mentha* spp. honey solvent extract with dichloromethane; C - bee-stomach solvent extract with dichloromethane; RI = retention indices on HP-5MS column; - = not identified; Min. = minimal percentage; Max. = maximal percentage; Av. = average percentage; SD. = standard deviation; ^a^ – identification confirmed with reference compound; ^*^ - tentatively identified; ^**^ - correct isomer not identified.

**Figure 2:**
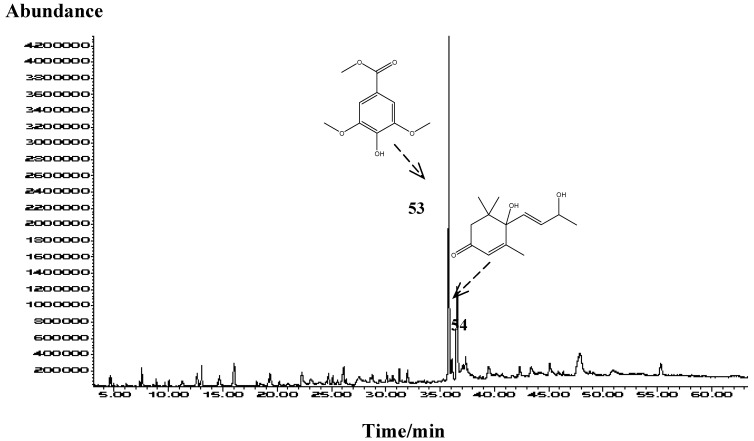
Representative TIC chromatogram of organic extractives obtained with the mixture of pentane and diethyl ether (1:2, v/v) of *Mentha* spp. honey.

Methyl syringate was the most abundant compound of all *Mentha* ssp. honey solvent extracts (Table 1, Figure 2) with minor percentage differences among different solvents: 47.9–56.2% (solvent pentane- diethyl ether 1:2 v/v); 38.3-48.6% (solvent dichloromethane). Methyl syringate was detected also in robinia, rape, chestnut, clover, linden blossom, dandelion, sunflower, thyme, manuka and fir honeys [20], but only in asphodel honey it reached the highest level [21]. Benzoic acid derivatives can be produced through the shikimate pathway [22], and methyl syringate [23,24] and its glucoside [25] were found in several plant extracts, but syringic acid methyl ester is also a typical laccase substrate in mediated breakdown of lignocellulose. Although methyl syrigate was considered previously [17] to be probably absorbed into the lipophilic honey from propolis of linden honey, preliminary research of the propolis composition gathered from the combs containing *Mentha* spp. honey (unpublished data) confirmed its absence in the collected propolis. In addition, it was not identified among the volatiles of propolis gathered from different region in Croatia [26]. Since methyl syringate was found in the corresponding bee-stomach extractive, it can be considered as phytochemical transferred to the honey. Among other shikimate pathway derivatives found (Table 1) the following compounds were the most abundant: phenylacetic acid (0.3–7.7%), methyl vanillate (0.3–1.3%), vanillic acid (0.1–0.8%) and 4-hydroxybenzoic acid (0.0-0.9%). However, in comparison with other unifloral honeys neither of the previous aromatic acids can be emphasized as potential marker of the botanical origin. Vomifoliol was the second abundant compound in the solvent extracts (7.0–26.6%), the most widespread 3,5,5-trimethylcyclohex-2-ene derivative (carotenoid degraded-like structure) in the honey samples. This compound probably arises through degradation of abscissic acid, a well-known growth hormone. The origin of degraded carotenoid-like structures in different honeys was already connected with the corresponding botanical sources [27]. In addition, vomifoliol was found in the extractive of bee-stomach. Another single abundant compound in the honey extracts was 3-hydroxy-4-phenyl-butan-2-one (0.6–1.9%). Furan derivatives were also found with minor percentages [5-hydroxymethylfurfural (0.1–5.1%), 2-furanmethanol (0.0–0.2%) and methyl 2-furoate (0.1–0.9%)], not as the honey markers but as indicators of absence of heat treatment and appropriate storage conditions. 

Besides methyl syringate, vomifoliol and 3,7-dimethylocta-1,5-dien-3,7-diol common compounds found in the honey and the bee-stomach were fatty acids, alcohols and carbonyls. Their presence was expected, not only because they are present in the bee-stomach, but also in the beeswax and therefore they cannot be considered important for botanical origin determination [28].

### 2.2. Organic Extractives Isolated by Headspace Solid-Phase Microextraction from the Honey

Terpenes were the most abundant compounds of *Mentha* spp. honey headspace (Table 2). The origin of terpenes and derivatives in the honey is usually associated to the floral nectar gathered by the honeybees. 

**Table 2:** *Mentha* spp. honey solvent-free organic extractives composition isolated by HS-SPME.

No.	Compound	RI	Area percentage (%)			
			Min.	Max.	Av.	SD.
1.	2-Furancarboxaldehyde	< 900	0.0	2.1	1.07	1.05
2.	3-Methylbutanoic acid	< 900	0.0	1.4	0.57	0.74
3.	2,5-Diethyltetrahydrofuran^*^	902	0.0	2.0	0.97	1.00
4.	1-(2-Furanyl)-ethanone	914	0.0	0.4	0.23	0.21
5.	Benzaldehyde^a^	965	0.0	0.8	0.47	0.42
6.	Hexanoic acid^a^	974	0.0	0.3	0.13	0.15
7.	(*Z*)-Hex-3-enoic acid	1013	0.0	1.0	0.43	0.51
8.	Benzyl alcohol^a^	1037	0.7	1.0	0.83	0.15
9.	Phenylacetaldehyde^a^	1048	0.8	1.3	1.07	0.25
10.	*trans*-Linalool oxide (furan type)	1076	1.8	2.8	2.37	0.51
11.	*cis*-Linalool oxide (furan type)	1091	0.9	1.9	1.43	0.50
12.	Linalool^a^	1101	1.0	3.1	1.80	1.14
13.	Hotrienol	1106	31.1	38.5	33.90	4.01
14.	2-Phenylethanol^a^	1116	1.0	2.8	1.73	0.95
15.	α-Isophorone	1124	0.0	0.6	0.27	0.31
16.	*p*-Mentha-1,5,8-triene^**^	1138	0.0	0.5	0.23	0.25
17.	Lilac aldehyde (isomer I)^**^	1159	0.0	0.5	0.23	0.25
18.	Neroloxide	1162	0.9	1.9	1.37	0.50
19.	Epoxylinalool	1178	0.0	0.3	0.13	0.15
20.	Benzoic acid^a^	1162	0.0	0.9	0.40	0.46
21.	Octanoic acid^a^	1174	0.0	1.6	0.63	0.85
22.	3,7-Dimethylocta-1,5-dien-3,7-diol	1191	0.0	4.7	2.13	2.38
23.	2-Methoxy-4-methylphenol	1199	0.5	6.0	2.73	2.89
24.	5-Hydroxymethylfurfural^a^	1230	0.0	1.0	0.53	0.50
25.	Phenylacetic acid^a^	1269	0.0	0.8	0.47	0.42
26.	Nonanoic acid^a^	1273	0.0	2.0	0.93	1.00
27.	2-Acetylanilline	1308	0.0	2.6	1.13	1.33
28.	4-Vinyl-2-methoxyphenol	1322	0.0	0.9	0.57	0.49
29.	3-Hydroxy-4-phenyl-butan-2-one	1354	1.0	2.5	1.63	0.78
30.	*cis*-Isoeugenol	1366	0.0	0.5	0.20	0.26
31.	Decanoic acid^a^	1370	0.0	0.5	0.20	0.26
32.	*trans*-β-Damascenone	1388	0.0	1.5	0.67	0.76
33.	Methyl vanillate^a^	1527	0.0	0.9	0.47	0.45
34.	Methyl syringate^a^	1744	1.0	2.4	1.87	0.76
35.	Nonadecane^a^	1900	0.0	0.6	0.27	0.31
36.	Hexadecanoic acid^a^	1963	1.0	2.1	1.60	0.56

RI = retention indices on HP-5MS column; - = not identified; Min. = minimal percentage; Max. = maximal percentage; Av. = average percentage; SD. = standard deviation; ^a^ – identification confirmed with reference compound; ^*^ - tentatively identified; ^**^ - correct isomer not identified.

**Figure 3:**
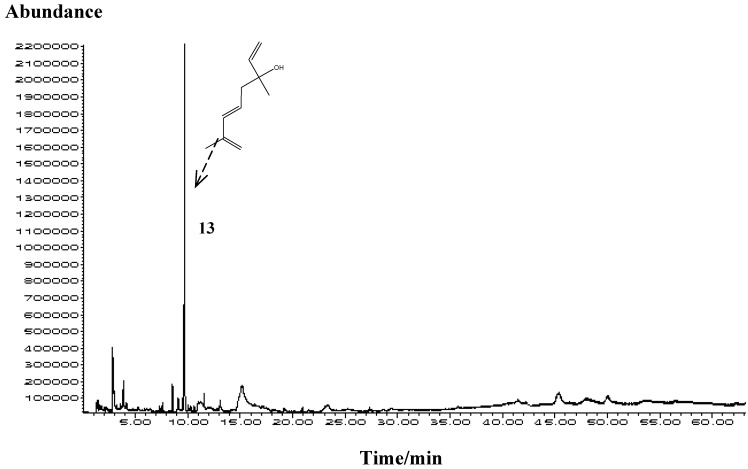
Representative TIC chromatogram of solvent-free extractives of *Mentha* spp. honey obtained by HS-SPME.

The major honey headspace compound was hotrienol (31.1%–38.5%) followed by *cis*- and *trans*-linalool oxides (0.9–2.8%), linalool (1.0–3.1%) and neroloxide (0.9–1.9%). A representative chromatogram of solvent-free organic extractives obtained by HS-SPME is presented in Figure 3. Hotrienol is known to be a thermally generated product, but the honey samples were not heated and therefore found hotrienol cannot be considered a thermally generated artifact in this case. These findings support a natural occurrence of hotrienol in non-thermally treated honey. Much lower proportions of hotrienol were found in unripe than in ripe honey, suggesting that hotrienol is probably formed during honey ripening [29] within the hive conditions (temperature, pH, enzymes that can lead to the oxidative degradation of linalool or the cleavage of the glycosidic bonds). Hotrienol can be derived either from 2,6-dimethyl-3,7-octadiene-2,6-diol (or its glycoconjugate form), from the allylic rearrangement of 3,7-dimethyl-1,7-octadiene-3,6-diol, or from dehydratation of 8-hydroxylinalool [30].

Hotrienol has been reported as a constituent of different honeys and therefore cannot be considered specific for *Mentha* spp. honey. However its high percentage in the headspace is a notable characteristic of this particular honey. Hotrienol, although present in honeys of various floral origins has been markedly detected in lavander honeys [31]. Benzaldehyde (0.0–0.8%), benzyl alcohol (0.7–1.0%), phenylacetaldehyde (0.8–1.3%), 2-phenylethanol (1.0–2.8%) and 3-hydroxy-4-phenylbutan-2-one (1.0–2.5%) were the most abundant benzene derivatives in the headspace. Methyl syringate (1.0–2.4%) and hexadecanoic acid (1.0–2.1%) were found with low percentages, as was expected. 

## 3. Experimental

### 3.1. Honey Samples and Bee-Stomach

Six *Mentha* spp. honey samples were investigated. The samples were obtained from professional beekeepers and no mechanical treatment or heat was used. The combs were placed in the area of wild growing *Mentha* spp. dominated by *Mentha aquatica* L. and *Mentha pulegium* L. Melissopalynological analysis was performed by the methods recommended by the International Commission for Bee Botany [32]. Microscopical examination was carried out on a Hund h 500 (Wetzlar, Germany) light microscope attached to a digital camera (Motic m 1000) and coupled to an image analysis system (Motic Images Plus software) for morphometry of pollen grains. Each sample was examined to determine the percentage of pollen grains. All the samples were stored in hermetically closed glass bottles at 4 °C until the volatiles isolation.

During the *Mentha* spp. honey flow the samples of returning foragers were collected. The bees were frozen in the field by liquid nitrogen and were stored in deep-freezer until their honey-sac contents was analyzed. After thawing, the abdomen of 70 bees was dissected by peeling off the tergit with forceps in order to expose the honey sac. The honey sac was removed and placed on a microscope slide. In each honey sac content pollen grain types were determined, and the honey sacs containing *Mentha* spp. pollen grains were frozen. After freezing, the entire content of analyzed honey sacs was pooled and put in a glass tube at 4 °C until the volatiles isolation.

### 3.2. Ultrasonic Solvent Extraction (USE)

Ultrasound-assisted solvent extraction (USE) was performed in an ultrasound cleaning bath (Elmasonic Typ S 30 H, Germany) by the indirect sonication mode (sweep mode), at the frequency of 37 kHz at 25 ± 3 °C. Forty grams of each *Mentha* spp. honey sample was dissolved in distilled water (22 mL) in a 100-mL flask. Magnesium sulfate (1.5 g) was added and each sample was extensively vortexed. A mixture of pentane-diethyl ether (1:2, v/v) and dichloromethane were separately used as the extraction solvents for each honey sample. Sonication was maintained for 30 min. After sonication, the organic layer was separated by centrifugation and filtered over anhydrous MgSO_4_. The aqueous layer was returned to the flask and another batch of the same extraction solvent (20 mL) was added and extracted by ultrasound for 30 min. The organic layer was separated in the same way as the previous one and filtered over anhydrous MgSO_4_, and the aqueous layer was sonicated a third time for 30 min with another batch (20 mL) of the extraction solvent. Combined organic extracts were concentrated to 0.2 mL by distillation with a Vigreaux column, and 1 μL was used for GC and GC/MS analyses. The content of honey-bee stomach (35 mg) was dissolved in distilled water (0.5 mL) in a 10 mL flask, magnesium sulfate (0.03 mg) was added and the sample was extensively vortexed. USE was performed using dichloromethane (3 mL) applying the same experimental procedure as described for the honey extraction (three-time extraction).

### 3.3. Headspace Solid-Phase Microextraction (HS-SPME)

The isolation of headspace volatiles was performed using a manual SPME fiber with a layer of polydimethylsiloxane/divinylbenzene (PDMS/DVB) obtained from Supelco Co (Bellefonte, PA, USA). The fiber was conditioned prior to use according to the manufacturer’s instructions. For HS-SPME extraction, honey/saturated water solution (5 mL, 1:1 v/v; saturated with NaCl) was placed in a 15 mL glass vial and hermetically sealed with PTFE/silicone septa. The vial was maintained in a water bath at 60 °C during equilibration (15 min) and extraction (45 min) and was partially submerged so that the liquid phase of the sample was below the water level. All the experiments were performed under constant stirring (1,000 rpm) with a magnetic stirrer. After sampling, the SPME fiber was withdrawn into the needle, removed from the vial, and inserted into the injector (250 °C) of the GC and GC-MS for 6 min where the extracted volatiles were thermally desorbed directly to the GC column.

### 3.4. Gas Chromatography and Mass Spectrometry (GC, GC/MS)

Gas chromatography analyses were performed on an Agilent Technologies (Palo Alto, CA, USA) gas chromatograph model 7890A equipped with flame ionization detector, mass selective detector, model 5975C and capillary column HP-5MS [(5%-phenyl)-methylpolysiloxane Agilent J & W GC column, 30 m, 0.25 mm i.d., coating thickness 0.25 μm]. Chromatographic conditions were as follows: helium was carrier gas at 1 mL·min^−1^, injector temperature was 250 °C, and FID detector temperature was 300 °C. HP-5MS column temperature was programmed at 70 °C isothermal for 2 min, and then increased to 200 °C at a rate of 3 °C·min^−1^ and held isothermal for 18 min. The injected volume was 1 μL and the split ratio was 1:50. MS conditions were: ionization voltage 70 eV; ion source temperature 230 °C; mass scan range: 30–300 mass units. The analyses were carried out in duplicate.

### 3.5. Data Analysis and Data Evaluation

The individual peaks were identified by comparison of their retention indices (relative to C_9_-C_25_ *n-*alkanes for HP-5MS) to those of authentic samples and literature [33], as well as by comparing their mass spectra with the Wiley 275 MS library (Wiley, New York, USA) and NIST 02 (Gaithersburg, MD, USA) mass spectral databases. The percentage composition of the samples was computed from the GC peak areas using the normalization method (without correction factors). The component percentages (Table 1 and Table 2) were calculated as mean values from duplicate GC and GC-MS analyses.

## 4. Conclusions

The chemical constituents of the organic extractives from *Mentha* spp. honey, headspace and the corresponding bee-stomach revealed the common compounds methyl syringate, vomifoliol and 3,7-dimethylocta-1,5-dien-3,7-diol (terpendiol I) as well as hotrienol that can be related to the plant origin and used for the characterization of this honey. Methyl syringate was the most abundant compound of the honey solvent extractives, followed by vomifoliol. The major honey headspace compounds were hotrienol, *cis*- and *trans*-linalool oxides, linalool and neroloxide Comparison of the honey organic extractives with the corresponding bee-stomach indicated that methyl syringate and vomofoliol were transferred to the honey, while terpendiol I was partially transformed to hotrienol in ripened honey.
